# Prostate cancer genes associated with *TMPRSS2–ERG* gene fusion and prognostic of biochemical recurrence in multiple cohorts

**DOI:** 10.1038/sj.bjc.6605519

**Published:** 2010-01-12

**Authors:** B G Barwick, M Abramovitz, M Kodani, C S Moreno, R Nam, W Tang, M Bouzyk, A Seth, B Leyland-Jones

**Affiliations:** 1 Emory Biomarker Service Center, Emory University, 1365C Clifton Road, NE, Atlanta, GA 30322, USA; 2 VM Institute of Research, 2020 University St., Suite 2040, Montreal, Quebec, Canada H3A 2A5; 3 Department of Pathology and Laboratory Medicine, Emory University School of Medicine, Atlanta, GA 30322, USA; 4 Divisions of Urology and Surgical Oncology, Sunnybrook Health Sciences Centre, Toronto, Ontario, Canada; 5 Sunnybrook Research Institute, University of Toronto, Ontario, Canada; 6 Robert W Woodruff Health Sciences Center, Winship Cancer Institute, Emory University School of Medicine, 1365C Clifton Road, NE, Suite 4014, Atlanta, GA 30322, USA; 7 Department of Human Genetics, Emory University School of Medicine, Atlanta, GA 30322, USA

**Keywords:** prostate cancer, TMPRSS2–ERG fusion, DASL, microarray, recurrence

## Abstract

**Background:**

Recent studies have indicated that prostate cancer patients with the *TMPRSS2–ERG* gene fusion have a higher risk of recurrence. To identify markers associated with *TMPRSS2–ERG* fusion and prognostic of biochemical recurrence, we analysed a cohort of 139 men with prostate cancer for 502 molecular markers.

**Methods:**

RNA from radical prostatectomy tumour specimens was analysed using cDNA-mediated, annealing, selection, extension and ligation (DASL) to determine mRNAs associated with *TMPRSS2–ERG* T1/E4 fusion and prognostic of biochemical recurrence. Differentially expressed mRNAs in T1/E4-positive tumours were determined using significance analysis of microarrays (false discovery rate (FDR) <5%). Univariate and multivariate Cox regression determined genes, gene signatures and clinical factors prognostic of recurrence (*P*-value <0.05, log–rank test). Analysis of two prostate microarray studies (GSE1065 and GSE8402) validated the findings.

**Results:**

In the 139 patients from this study and from a 455-patient Swedish cohort, 15 genes in common were differentially regulated in T1/E4 fusion-positive tumours (FDR <0.05). The most significant mRNAs in both cohorts coded ERG. Nine genes were found prognostic of recurrence in this study and in a 596-patient Minnesota cohort. A molecular recurrence score was significant in prognosticating recurrence (*P*-value 0.000167) and remained significant in multivariate analysis of a mixed clinical model considering Gleason score and TMPRSS2–ERG fusion status.

**Conclusions:**

TMPRSS2–ERG T1/E4 fusion-positive tumours had differentially regulated mRNAs observed in multiple studies, the most significant one coded for ERG. Several mRNAs were consistently associated with biochemical recurrence and have potential clinical utility but will require further validation for successful translation.

Prostate cancer prognosis after radical prostatectomy is incompletely assessed by clinical markers such as Gleason score, tumour, node, metastasis (TNM) stage, surgical margin status and preoperative prostate-specific antigen (PSA) level. Recently identified genetic markers could provide clinical utility for prostate cancer progression and recurrence independent of the current clinical markers, thereby improving patient management. Examples of such markers include the E twenty-six (ETS) family of transcription factors that were identified through outlier profile analysis that detected an elevated expression of ETS factors (Tomlins *et al*, 2005). Normal and benign prostate tissues, as well as prostatic intraepithelial neoplasia lesions, lack the expression of ERG, whereas expression of *TMPRSS2–ERG* fusion mRNAs occurs uniquely in prostate adenocarcinoma because of the fusion of a promoter/enhancer region of an androgen-responsive prostate-specific serine protease 2-encoding gene, *TMPRSS2*, to the v-ets erythroblastosis virus E26 oncogene homologue, *ERG*, or the ETS variant 1 gene, *ETV1* (Tomlins *et al*, 2005; Narod *et al*, 2008), and over 20 other gene fusion variants (Perner *et al*, 2006; Mehra *et al*, 2007; Tu *et al*, 2007). Fusion of *TMPRSS2* (21q22.2) and *ERG* (21q22.3), which are within 3 Mb of each other, results from a chromosome 21 microdeletion in approximately two-thirds of fusion cases (Yoshimoto *et al*, 2006b). The most common variant is a recombination between exon 1 of *TMPRSS2* and exon 4 of *ERG*, designated T1/E4 (Clark *et al*, 2007; Jhavar *et al*, 2008), and some studies indicate that this variant accounts for 85% of reported fusions (Wang *et al*, 2006; Mehra *et al*, 2007).

Both the prevalence and prognostic significance of the *TMPRSS2*–*ERG* T1/E4 fusion have been examined in multiple studies with some discrepancy in results. Although *TMPRSS2–ERG* fusion has typically been reported as prevalent in 40–50% of prostate tumours, the range has varied by as much as 25–60% (Nam *et al*, 2007; Setlur *et al*, 2008; Sun *et al*, 2008; Yoshimoto *et al*, 2008; Hofer *et al*, 2009; Mosquera *et al*, 2009). The techniques used for *TMPRSS2–ERG* detection, novel potential fusion products and genetic differences in population cohorts may account for these discrepancies. This is highlighted by the finding of Mosquera *et al* (2009) that Caucasian populations were less likely to have the *TMPRSS2–ERG* fusion and this could explain the 22.6% prevalence rate observed by Setlur *et al* (2008) in a Swedish cohort of 455 prostate cancer cases. Irrespective of the cause for reported variation in prevalence, further studies are needed to clearly define the prevalence rate of *TMPRSS2–ERG* fusions in the context of population genetics.

Commensurate with the range in prevalence of *TMPRSS2–ERG* fusions is the variation in reported prognostic significance. Several studies have indicated that *TMPRSS2–ERG* fusions confer a worse prognosis (Nam *et al*, 2007; Attard *et al*, 2008a; Clark *et al*, 2008), whereas others have not (Gopalan *et al*, 2009). Discrepancies in the reported prognostic significance of *TMPRSS2–ERG* fusions can be explained in a similar manner by factors affecting the variation in prevalence of *TMPRSS2–ERG* (i.e., cohort race/ethnicity, fusion detection technique), and are also liable to the primary end point of the study (i.e., biochemical recurrence, overall survival). The complexity contributing to the discordant prognostic significance of *TMPRSS2–ERG* fusions was mentioned by FitzGerald *et al* (2008), who reported that *TMPRSS2–ERG* fusions did not result in reduced survival, but that the combination of *TMPRSS2–ERG* fusion and amplification of the fusion gene conferred a worse prognosis. As in the case of *TMPRSS2–ERG* fusion prevalence, to properly understand the prognostic significance of *TMPRSS2–ERG* fusions, further standardised studies are needed. Proper patient tracking and characterisation in combination with proper sample management can ensure accurate molecular characterisation and spur translation of clinical tools.

In an effort to better characterise the molecular implications of *TMPRSS2–ERG* fusions in prostate cancer and to identify other genetic prognostic markers, we have utilised RNA extracted from radical prostatectomy specimens in a prospective cohort previously characterised for *TMPRSS2–ERG* T1/E4 fusion transcript expression by qualitative PCR (Nam *et al*, 2007). This cohort (hereafter referred to as the Toronto cohort) of 165 patients had 81 (49%) fusion-positive tumours in which patients with a detectable fusion transcript had a significantly higher risk of biochemical recurrence (58% at 5 years) than did patients whose tumours did not express detectable fusion transcripts (8% at 5 years). Therefore, *TMPRSS2–ERG* T1/E4 fusion was found to be a strong prognostic factor independent of grade, stage and PSA level in this cohort (Nam *et al*, 2007). We have subsequently made use of RNA from 139 of the 165 patients and characterised the expression of 502 cancer-related genes using the cDNA-mediated, annealing, selection, extension and ligation (DASL) assay.

## Materials and methods

### RNA samples

Total RNA samples from frozen prostate tumour specimens used in this study were prepared previously (Nam *et al*, 2007). Aliquoted RNA samples were shipped on dry ice to the Emory Biomarker Service Center (EBSC), Emory University, Atlanta, GA, USA, for use in the DASL assay. RNA concentration was quantified by a Nanodrop spectrophotometer (Wilmington, DE, USA) and quality was assessed using the Agilent Bioanalyzer (Foster City, CA, USA) for which RNA integrity number of >7 was used as a quality criteria.

### DASL assay performance, reproducibility and data normalisation

The DASL assay was performed on Illumina's 502-gene Human Cancer Panel using 200 ng of input RNA. The manufacturer's instructions were followed without any changes. Samples were hybridised on two Universal Array Matrices (UAMs). Hybridised UAMs were scanned using the BeadStation 500 Instrument (Illumina, San Diego, CA, USA). Data were interpreted and quantile normalised using GenomeStudio v1.0.2 (Illumina). Experimental replicates (same RNA assayed twice) were assessed for reproducibility and subsequently averaged so as to represent each patient's tumour sample with one gene expression profile.

### Data analysis and meta-analysis

Differential mRNA expression of *TMPRSS2–ERG* T1/E4 fusion-positive *vs* fusion-negative tumours was assessed using significance analysis of microarrays (Tusher *et al*, 2001) for which 1000 random class assignment permutations estimated a false discovery rate (FDR) ⩽5%. Hierarchical clustering was generated in R using the heatmap.2 package in which distance was computed using a Euclidean dissimilarity metric with an average-linkage clustering algorithm. Data were displayed with mRNA intensity *z*-score normalised. Gene Ontology analysis was conducted using the R package GOstats with a significance value of *P*<0.01 of over-representation computed by the hypergeometric test using the lumiHumanAll.db annotation file. Univariate Cox proportional hazards regression was conducted in R using the Cox proportional hazards survival package (CoxPH) and was conducted on each gene expression profile and clinical factor independently. Multivariate Cox analysis considered the clinical factors that were significant (*P*<0.05) in univariate analysis, as well as a recurrence predictor built as a weighted average of the expression level of genes, which were significant in univariate analysis in both the Toronto data set and that from Nakagawa *et al* (2008). Kaplan–Meier curves were generated in R using the survival package, and significance testing utilised the survdiff function for which the log–rank test determined the *P*-value. Analysis utilised expression profiles from studies by both Setlur *et al* (2008) and Nakagawa *et al* (2008), which were downloaded from Gene Expression Omnibus (GEO; http://www.ncbi.nlm.nih.gov/geo/) and had the series numbers GSE8402 and GSE10645, respectively. The same differential annotation and prognostic analyses methods described above were used on the meta-analysis sets.

## Results

**Figure 1:**
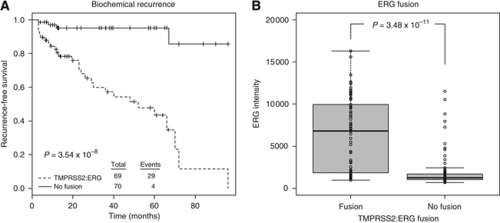
Characteristics of prostate cancer patients with and without the T1/E4 TMPRSS2–ERG fusion. (**A**) Patients with TMPRSS2–ERG fusion-positive tumours experienced a higher rate of biochemical recurrence opposed to those who did not have the gene fusion (*P*-value 3.54 × 10^−8^, log–rank test). (**B**) ERG expression was upregulated in TMPRSS2–ERG fusion-positive tumours by 3.07-fold (*P*-value 3.48 × 10^−11^, Student's *t*-test).

After RNA and assay quality control, 139 patient tumours were characterised on the DASL assay for 502 cancer-related genes (GEO series GSE18655). Seven samples were run as experimental replicates to estimate assay reproducibility for which an average Pearson's *R*^2^ of 0.965 indicated highly reproducible data (Supplementary Figure 1). Moreover, unsupervised hierarchical clustering of all samples and probes resulted in experimental replicates clustering together without exception (Supplementary Figure 2). The Toronto cohort, a subset of that previously characterised for clinical markers (Nam *et al*, 2007), includes 69 patients with *TMPRSS2–ERG* T1/E4 fusion-positive tumours and 70 prostate tumours that were *TMPRSS2–ERG* fusion negative. Fusion status indicated a significantly worse outcome with respect to biochemical recurrence (Figure 1A, *P*-value 3.54 × 10^−8^ log–rank test) similar to that observed in the entire cohort (Nam *et al*, 2007). As previously reported, patients with *TMPRSS2–ERG* fusion-positive tumours had a significantly higher expression of *ERG* transcripts (Figure 1B, *P*-value 3.48 × 10^−11^, two-sided Student's *t*-test), which is most likely a result of androgen-responsive promoter elements in *TMPRSS2* driving expression (Tomlins *et al*, 2005). Overexpression of *ERG* was validated using reverse transcriptase PCR, which corroborated the *ERG* overexpression found by microarray results (Supplementary Figure 3, *P*-value 8.13 × 10^−10^, two-sided Student's *t*-test).

**Figure 2:**
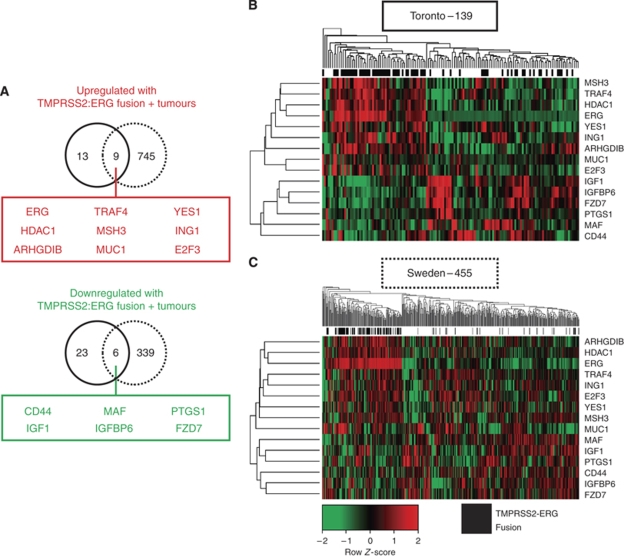
Validated genes differentially expressed in *TMPRSS2–ERG* fusion-positive tumours. (**A**) Significance testing of genes differentially regulated in *TMPRSS2–ERG* fusion-positive prostate tumours in the Toronto cohort of 139 patients characterised for 502 genes (solid black line) was validated in a Swedish cohort (Setlur *et al*, 2008) of 455 patients characterised for 6144 genes (dashed black line). Nine genes upregulated with the *TMPRSS2–ERG* fusion in both cohorts are shown at the top (red box), whereas six genes downregulated in both cohorts are shown at the bottom (green box). Hierarchical clustering of the 15 common differentially expressed genes segregates *TMPRSS2–ERG* fusion-positive tumours as indicated in black above the heatmaps and below the clustering dendrogram in (**B**) the Toronto cohort of 139 patients and (**C**) the Swedish cohort of 455 patients.

To investigate molecular biomarkers differentially regulated in *TMPRSS2–ERG* fusion-positive tumours, significance testing was conducted using significance analysis of microarrays (Tusher *et al*, 2001) for both the Toronto cohort and that of the 455-patient Swedish cohort (Setlur *et al*, 2008). Using an FDR ⩽5% yielded 51 genes differentially regulated in *TMPRSS2–ERG* fusion-positive tumours in the Toronto cohort (Supplementary Table 1). Nine upregulated genes and six downregulated genes were validated by replicating the analysis on the Swedish cohort (Setlur *et al*, 2008), which was characterised for expression of 6144 transcripts (Figure 2A, FDR <5%). In both the Toronto and Swedish cohorts, ERG was uniquely the most significant differentially regulated transcript in *TMPRSS2–ERG* fusion-positive tumours (Supplementary Figure 4). Genes annotated for mismatch base repair and histone deacetylation functions were over-represented in Gene Ontology analysis of common upregulated genes in T1/E4-positive tumours (Supplementary Table 2, *P*<0.01). Downregulated genes were over-represented for annotations that included the insulin-like growth factor (IGF) and Jak-Stat signalling pathways, suggesting that these pathways may be attenuated in T1/E4-positive tumours (Supplementary Table 2, *P*<0.01). Hierarchical clustering of tumour expression profiles across common differentially regulated genes resulted in segregation of *TMPRSS2–ERG* fusion-positive tumours (Figures 2B and C), suggesting that *TMPRSS2–ERG* fusion-positive tumours have a distinct molecular metabolism that is replicated in multiple cohorts.

**Figure 3:**
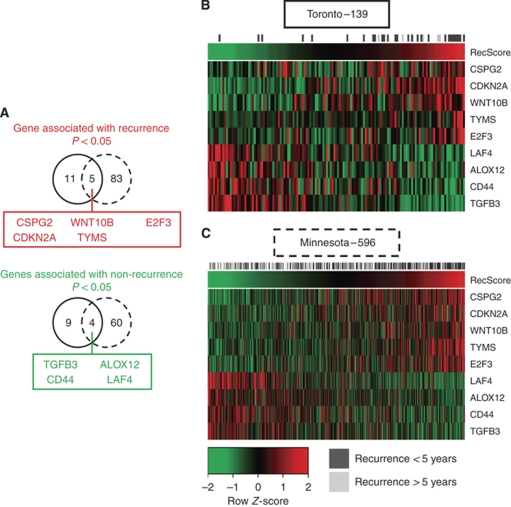
Common genes prognostic of biochemical recurrence. (**A**) Univariate Cox proportional hazards regression determined genes associated with biochemical recurrence in the Toronto cohort of 139 patients, and a Minnesota cohort of 596 patients (Nakagawa *et al*, 2008) identified seven genes in common, five associated with recurrence and two associated with non-recurrence. Supervised heatmaps ordered by the seven-gene expression recurrence score showed an increased incidence of recurrence with increased recurrence score in (**B**) the Toronto cohort of 139 patients and (**C**) the Minnesota cohort of 596 patients (Nakagawa *et al*, 2008).

**Figure 4:**
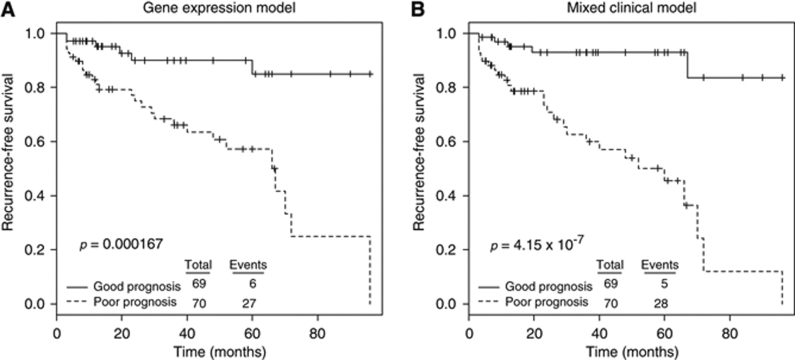
Kaplan–Meier survival analysis of the Toronto cohort. (**A**) Seven-gene expression recurrence score used to segregate patients into good and poor prognostic categories. (**B**) A mixed clinical model composed of the Gleason score, TMPRSS2–ERG fusion status and the seven-gene expression recurrence score is better able to prognosticate recurrence.

To determine the molecular factors associated with biochemical recurrence (defined as a PSA increase of ⩾0.2 ng ml^−1^ on at least two consecutive measurements that are at least 3 months apart), univariate Cox proportional hazards regression was conducted in the Toronto cohort and replicated in a 596-patient Minnesota cohort (Nakagawa *et al*, 2008). The Toronto data set yielded 16 genes associated with recurrence and 11 genes associated with non-recurrence (Supplementary Table 3, *P*<0.05). Repeating this analysis in the Minnesota cohort validated five genes associated with biochemical recurrence and four genes associated with non-recurrence (Figure 3A, *P*<0.05). Gene Ontology functional annotation of genes commonly associated with recurrence yielded over-representation of deoxyribosylthymine monophosphate biosynthesis, negative regulation of leukocyte activation – specifically T- and B-cell lymphocytes – and inhibition of cell-matrix adhesion. Conversely, annotation of genes associated with non-recurrence resulted in cell-matrix adhesion and collagen binding (Supplementary Table 4, *P*<0.01). Common genes prognostic of recurrence were used to build a recurrence score calculated as the sum product of the expression intensity of each gene by its Cox coefficient determined by regression analysis. Ordering samples by the recurrence score in a supervised heatmap produced a trend whereby patients who did not have recurrence were separated from those who did in both the Toronto and Swedish cohorts (Figures 3B and C). More importantly, the recurrence score was significant in univariate Cox regression and remained significant in a multivariate model considering clinical factors that were significant (*P*<0.05) in the univariate analysis, namely, preoperative PSA level, Gleason score and *TMPRSS2–ERG* fusion status (Table 1 – Toronto cohort). Furthermore, the nine-gene expression recurrence score was significantly associated with biochemical recurrence by itself (Figure 4A, *P*-value 0.000167) and in a multivariate model considering the Gleason score and *TMPRSS2–ERG* fusion status (i.e., those clinical data significant in univariate analysis; Figure 4B, *P*-value 4.15 × 10^−7^).

## Discussion

The previously identified *TMPRSS2–ERG* T1/E4 gene fusion represents a potentially important factor in prostate cancer progression, the prognostic value of which has yet to be fully defined. In this cohort, the *TMPRSS2–ERG* T1/E4 gene fusion is highly associated with biochemical recurrence and these findings have been supported by other studies (Clark *et al*, 2008; Attard *et al*, 2008b), although some have not found a worse prognosis conferred by fusion status (Gopalan *et al*, 2009). Also of primary clinical importance, and even less defined, is the potential predictive value of *TMPRSS2–ERG* fusion (i.e., is treatment course predicted by fusion status?). To these important prognostic and predictive end points, this study makes two significant contributions.

First, this study finds 27 genes prognostic of biochemical recurrence, with 16 genes associated with recurrence and 11 genes associated with non-recurrence. Replication of this analysis in a Minnesota cohort of 596 patients (Nakagawa *et al*, 2008) validated five genes associated with recurrence and four associated with non-recurrence (Figure 3A). Use of these nine genes to create a recurrence score yielded a highly significant prognostic variable that could be used to segregate patients into high- and low-risk categories. This nine-gene expression recurrence score remained significant in multivariate analysis considering clinical factors such as Gleason score and other genetic factors such as *TMPRSS2–ERG* fusion status. Although translation of any such prognostic test would require more analysis and validation, not only by reverse transcriptase PCR but also in other independent cohorts, this study contributes to the current body of knowledge with respect to identifying biomarkers with the potential to improve prostate cancer patient management. Furthermore, certain genes that we found prognostic of recurrence, which were not validated in the Minnesota cohort, have previously been linked to prostate cancer progression, such as mucin 1 (Cozzi *et al*, 2005), which has also been proposed as a candidate for targeted therapy (Li and Cozzi, 2007), and may warrant further consideration as a prognostic biomarker.

Second, this study finds 51 differentially expressed genes dependent on *TMPRSS2–ERG* T1/E4 fusion status, 15 (Supplementary Table 1, in bold) of which are validated in a 455-patient Swedish cohort (Setlur *et al*, 2008) using the same analysis. Functional annotation of the 15 genes replicated in both cohorts suggests that TMPRSS2–ERG fusion is associated with mismatch base repair and histone deacetylation molecular functions. It is therefore a reasonable hypothesis that therapeutic modalities targeting these pathways may be well suited for TMPRSS2–ERG fusion-positive prostate cancer patients. The histone deacetylation inhibitors SAHA (vorinostat) and LBH589, both currently being tested in clinical trials, can inhibit androgen receptor (AR)-mediated transcriptional activation of a number of genes including *TMPRSS2–ETS* fusion genes (Welsbie *et al*, 2009). Similarly, mismatch base repair has the potential to be targeted with the use of poly(ADP-ribose) polymerase inhibitors, a class of compounds showing significant potential in the treatment of hereditary (BRCA mutation) breast cancer and currently being tested in several clinical trials (Fong *et al*, 2009). Conversely, prostate cancer patients without the *TMPRSS2–ERG* fusion gene may be better suited for therapies that target IGFs and Jak-Stat signalling, in which IGF signalling can be targeted through the use of tyrosine kinase inhibitors or monoclonal antibodies. Finally, although not replicated in the Swedish cohort, we found attenuation of the potent tumour-suppressor phosphatase and tensin homologue, PTEN, in TMPRSS2–ERG fusion-positive tumours. Loss of PTEN expression has been strongly associated with prostate cancer progression (Koksal *et al*, 2004; Bertram *et al*, 2006; Yoshimoto *et al*, 2006a; Schmitz *et al*, 2007) and has recently been implicated as cooperating with TMPRSS2–ERG fusion in high-grade prostatic intraepithelial neoplasia (Carver *et al*, 2009; King *et al*, 2009). Loss of PTEN results in PI3K activation, which in turn can be targeted with emerging modalities inhibiting PI3K or PI3K and mTor such as SF1126 and BEZ235, respectively. This study supports other evidence that TMPRSS2–ERG fusion status may have important predictive implications of such therapies in the efficacious treatment of prostate cancer.

Our findings of several replicated mRNAs prognostic of recurrence indicate that there are consistent hallmarks of refractory disease that may have clinical utility. Furthermore, the TMPRSS2–ERG fusion may have an important prognostic and predictive value, because of which differentially regulated mRNAs (most notably ERG) in repeated experiments suggest that targeted therapy in prostate cancer could be aided by this biomarker. These hypotheses will require standardised clinical trials with proper patient tracking and sample handling to translate these molecular targets into meaningful clinical tools.

## Figures and Tables

**Table 1:** Clinical and molecular factors for the Toronto – 139

		***TMPRSS2–ERG* T1/E4 fusion**		**Recurrence model (*P*-value)**	
	**Total**	**Positive**	**Negative**	**Univariate**	**Multivariate**
Cohort size (*n*)	139	69	70	—	—
Biochemical recurrence	33	29	4	—	—
Average follow-up (months)	30.9	25.8	36	—	—
Average age (years)	61.7	61.1	62.2	0.0880	—
*Preoperative PSA (ng ml*^−*1*^)				**0.0210**	0.6200
Average	8.9	9.3	8.5		
Range	(2.2–43.0)	(3.4–38.9)	(2.2–43.0)		
*Gleason score*				**0.0190**	**0.0280**
5–6 (%)	38 (27.3)	19 (27.5)	19 (27.1)		
7 (%)	90 (64.7)	46 (66.7)	44 (62.9)		
8–9 (%)	11 (7.9)	4 (5.8)	2 (10.0)		
*Pathological stage*				0.0860	—
Organ confined (%)	59 (42.4)	29 (42.0)	30 (42.9)		
Extraprostatic extension (%)	70 (50.4)	35 (50.7)	35 (50.0)		
Seminal vesicle invasion (%)	10 (7.2)	5 (7.2)	5 (7.1)		
*Positive margin*				0.4000	—
No (%)	62 (44.6)	33 (47.8)	29 (41.4)		
Yes (%)	77 (55.4)	36 (52.2)	41 (58.6)		
TMPRSS2–ERG fusion	—	—	—	**0.0000085**	**0.0004**
Nine-gene recurrence score (95% CI)	2.01	3.37 (0.37, 7.18)	1.58 (−0.94, 4.25)	**0.0000002**	**0.0270**

Abbreviations: CI=confidence interval; PSA=prostate-specific antigen.

Cohort clinical characteristics for the 139 prostate cancer patients in the Toronto cohort are listed out for *TMPRSS2–ERG* T1/E4 fusion-positive and fusion-negative patients. Factors were assessed for their association with biochemical recurrence when relevant (indicated by a univariate *P*-value). Factors prognostic of recurrence (*P*<0.05) were used in a multivariate model of recurrence. Significant factors are indicated in bold. The nine-gene recurrence score (composed of the genes listed in Figure 3A) is composed of mRNAs replicated as prognostic of recurrence in this experiment and a 596-patient Minnesota experiment (Nakagawa *et al*, 2008).
